# Cingulate white matter mediates the effects of fecal *Ruminococcus* on neuropsychiatric symptoms in patients with amyloid-positive amnestic mild cognitive impairment

**DOI:** 10.1186/s12877-023-04417-9

**Published:** 2023-11-07

**Authors:** Chun-Che Hung, Yi-Ping Chao, Yejin Lee, Chi-Wei Huang, Shu-Hua Huang, Chiung-Chih Chang, Chia-Hsiung Cheng

**Affiliations:** 1 Department of Occupational Therapy and Graduate Institute of Behavioral Sciences, Chang Gung University, No. 259, Wenhua 1st Road, 333 Taoyuan, Taiwan; 2 Laboratory of Brain Imaging and Neural Dynamics (BIND Lab), Chang Gung University, Taoyuan, Taiwan; 3 Department of Computer Science and Information Engineering, Chang Gung University, Taoyuan, Taiwan; 4 Department of Otorhinolaryngology-Head and Neck Surgery, Chang Gung Memorial Hospital, Linkou, Taiwan; 5 Program in Occupational Therapy, Washington University School of Medicine, St. Louis, MO USA; 6 Department of Neurology, Institute for Translational Research in Biomedicine, Kaohsiung Chang Gung Memorial Hospital, Chang Gung university College of Medicine, No. 123 Ta-Pei Rd., Niau-Sung Dist, 833 Kaohsiung, Taiwan; 7 Department of Nuclear Medicine, Kaohsiung Chang Gung Memorial Hospital, Chang Gung University College of Medicine, Kaohsiung, Taiwan; 8 Healthy Aging Research Center, Chang Gung University, Taoyuan, Taiwan; 9 Department of Psychiatry, Chang Gung Memorial Hospital, Linkou, Taiwan

**Keywords:** Amnestic mild cognitive impairment, Diffusion tensor imaging, Gut microbiota, Limbic system, Neuropsychiatric symptoms, Ruminococcus

## Abstract

**Background:**

Microbiota-gut-brain axis interacts with one another to regulate brain functions. However, whether the impacts of gut dysbiosis on limbic white matter (WM) tracts contribute to the neuropsychiatric symptoms (NPS) in patients with amyloid-positive amnestic mild cognitive impairment (aMCI+), have not been explored yet. This study aimed to investigate the mediation effects of limbic WM integrity on the association between gut microbiota and NPS in patients with aMCI+.

**Methods:**

Twenty patients with aMCI + and 20 healthy controls (HCs) were enrolled. All subjects underwent neuropsychological assessments and their microbial compositions were characterized using 16S rRNA Miseq sequencing technique. Amyloid deposition inspected by positron emission tomography imaging and limbic WM tracts (i.e., fornix, cingulum, and uncinate fasciculus) detected by diffusion tensor imaging were additionally measured in patients with aMCI+. We employed a regression-based mediation analysis using Hayes’s PROCESS macro in this study.

**Results:**

The relative abundance of genera *Ruminococcus* and *Lactococcus* was significantly decreased in patients with aMCI + versus HCs. The relative abundance of *Ruminococcus* was negatively correlated with affective symptom cluster in the aMCI + group. Notably, this association was mediated by WM integrity of the left cingulate gyrus.

**Conclusions:**

Our findings suggest *Ruminococcus* as a potential target for the management of affective impairments in patients with aMCI+.

**Supplementary Information:**

The online version contains supplementary material available at 10.1186/s12877-023-04417-9.

## Introduction

Amnestic mild cognitive impairment (aMCI), mainly characterized by episodic memory deficits, is a prodromal phase of Alzheimer’s disease (AD) dementia [1]. The primary neuropathological hallmarks of AD are the deposition of β-amyloid plaques in the cerebral parenchyma, along with the presence of neurofibrillary tangles within neurons [2]. In recent years, the National Institute on Aging − Alzheimer’s Association has proposed a biomarker-based classification system, which categorizes the main AD biomarkers into three types, including amyloid (A), tau (T), and neurodegeneration (N) [3]. Notably, the category of amyloid-positivity (A+) accounts for most individuals with symptomatic AD.

In the stage of aMCI, cognitive impairments are often accompanied by the presence of neuropsychiatric symptoms (NPS), of which mood disturbances have been widely reported [4, 5]. Since NPS contribute to increased functional impairments and a greater burden on their caregivers, a better characterization of NPS in these patients is of particular importance [6]. To improve the disease management, Gauthier and colleagues have proposed that NPS clusters should be identified in clinical trials, which enables similar symptoms to be studied together, thereby strengthening results of exploring their pathogenesis and treatment [7]. Based on clinical observations, NPS can be categorized into three clusters: the psychotic cluster (i.e., hallucinations and delusions), the hyperactivity cluster (i.e., lability, disinhibition, agitation/aggression, and aberrant motor behavior), and the affective symptom cluster (i.e., apathy, depression, anxiety, and changes in sleep or appetite) [8].

Growing evidence demonstrates a complex bidirectional communication between central nervous system and gut microbiota, termed the microbiota-gut-brain (MGB) axis [9]. This reciprocal interaction is implicated in brain development and function mediated by different biochemical signaling pathways such as neurotransmitters and hypothalamic-pituitary-adrenal (HPA) axis [10]. Dysfunction of the MGB axis has been found to be closely involved in the pathogenesis of NPS [11, 12]. It has been reported that the disruption of gut homeostasis influenced the release of monoamine neurotransmitters and stimulated inflammation in the brain through the systemic circulation, which in turn, impacted mood and behavior [13]. Likewise, gut dysbiosis-triggered HPA axis dysregulation led to reduced expression of brain-derived nerve growth factor and mental health problems including anxiety and depression [14]. Several bacterial strains have been found to be related to NPS in AD. For instance, genera *Anaerobacterium* and *Taibaiella* were negatively correlated with depression, whereas the association of anxiety with class *Cytophagia* and its order *Cytophagales* showed the opposite patterns [15]. Nonetheless, the mechanisms underlying these relationships are not be fully elucidated. Exploration of this issue may provide clinical insights by manifesting the MGB axis as potential targets for effective management of NPS.

The limbic system, a group of interconnected cortical and subcortical structures, plays critical roles in episodic memory and emotional processing [16]. Papez proposed the first unified limbic network model for linking action and perception to emotion [17]. A revised model proposed by Catani and colleagues offered a more comprehensive understanding of the limbic system [16]. Based on the Catani’s model, these tracts were impaired early in the patients with aMCI [18]. Since white matter (WM) loss commonly precedes gray matter atrophy, it is useful to apply diffusion tensor imaging (DTI) to measure the WM changes in patients with aMCI [19]. Fractional anisotropy (FA), mean diffusivity (MD), axial diffusivity, and radial diffusivity (RD) are the widely used and representative diffusion metrics. Furthermore, tract-based spatial statistics (TBSS) is a hypothesis-free approach that generates a WM skeleton to facilitate voxel-based analysis in WM pathways across individuals and groups [20]. Using the TBSS method, more severity of apathy was associated with lower FA values of multiple WM tracts in patients with aMCI [21]. Previous research has also shown that individuals with coexisting aMCI and late-life depression manifested widespread limbic degradation [22]. From the perspective of the MGB axis, the limbic system is chiefly involved in gut control. Existing evidence has revealed that the signaling from gut microbiota was transduced to the limbic system through the HPA axis and enteric nervous system. Additionally, NPS were found to be associated with the fluctuation of neurotransmitters because some gut microbial products acted as “neuro-nucleo-modulins” and thereby led to the host’s limbic degradation [23, 24]. Therefore, the limbic region of the mammalian brain is linked to both the internal and external homeostasis of the organism [25]. These findings suggest that the limbic structure is likely a convergence hub for the overlapping effects of gut dysbiosis and NPS.

Major advances in non-invasive neuroimaging techniques enable us to investigate the role of gut microbiota in brain function and behavior, which can foster the identification of potential mediators of this relationship. Specifically, Cai and colleagues showed that the interplay between alpha diversity and behaviors was mediated by functional connectivity of several brain regions in healthy young adults [26]. Recent evidence also demonstrated that a higher level of anxiety was linked to a smaller gray matter volume of the right dorsolateral prefrontal cortex and reduced Chao1 in healthy subjects [27]. In individuals with obesity, there was a potential relationship among the relative abundance of phylum *Actinobacteria*, WM microstructure, and cognitive functions [28]. Persistent probiotic ingestion led to a decrease in activity of brain areas involved in emotional regulation when subjects were administered with negative facial expressions, indicating that alterations of the gut microbiota with probiotics had a measurable effect on the brain [29]. Nevertheless, it has not been investigated, so far, the impact of gut dysbiosis on brain WM integrity in patients with aMCI.

In this study, we compared gut microbial diversity and composition using 16S rRNA gene sequencing between patients with amyloid-positive aMCI (aMCI+) and their healthy counterparts. The limbic WM integrity was analyzed by high-resolution DTI data. In the aMCI + group, Neuropsychiatric Inventory (NPI) was employed to assess their severities of NPS. We focused on NPS due to their close relationships with gut microbiota and the corresponding metabolites [30, 31]. We hypothesized that gut dysbiosis would be linked to limbic WM degeneration, which could mediate the association between the dysbiotic microbiota and NPS.

## Materials and methods

### Participants

This study was approved by the ethics committee of the Chang Gung Memorial Hospital, Taiwan (202001151B0). All participants were required to provide written informed consent before participating in this study. A total of 20 individuals with aMCI + and 20 healthy controls (HC) without cognitive problems, mostly patients’ spouses, were recruited from the Department of Neurology of Kaohsiung Chang Gung Memorial Hospital. Each subject underwent detailed clinical and neuropsychological assessments. Clinical diagnosis of aMCI was based on the Petersen’s criteria [32]. In addition, aMCI + patients were administered with blood sample tests, amyloid burden evaluation, and DTI scans. The Centiloid scale was applied to standardize quantitative amyloid measures by the neurologists independently and blind to patients’ clinical information (Supplementary methods) [33, 34]. We also provided each participant with sterile plastic containers to collect a fresh fecal sample at home.

Exclusion criteria for both HC and aMCI + groups included: (1) evidence of psychiatric or other neurological disorders; (2) received antibiotics, prebiotics, or probiotics in past two months; (3) suffered drug or alcohol additions; (4) suffered irritable bowel syndrome or inflammatory bowel diseases within one year; (5) had combined serious heart, liver, kidney or hematopoietic system disorders; (6) with visual, auditory, or motor dysfunctions influencing cognitive performance.

#### Assessments of neuropsychiatric symptoms

The NPI is a retrospective (up to 1 month) 12-item questionnaire to evaluate the presence and severity of NPS in neurodegenerative disorders [4]. A total score is the sum of each NPI item, obtained by the frequency (from 1 = less than once per week to 4 = once or more per day) × severity (from 1 = mild to 3 = severe) rating in each item. Furthermore, the sum of scores from hallucinations and delusions represented the psychotic composite score; the sum of scores from aberrant motor behavior, aggressiveness, irritability, and disinhibition represented the behavioral composite score; and the sum of scores from depression, apathy, anxiety, sleep, and appetite represented the affective composite score [8].

### Fecal sample collection and gut microbiota analysis

Fecal samples were collected from each participant at home using sterile containers. The participants returned the containers by overnight delivery and chilled them with frozen gel packs. These containers were stored at -80 °C until processing. Microbial DNA in each stool sample was extracted using the CatchGene Stool DNA Kit (CatchGene Co., Ltd., Taiwan) from 100 mg sample. The procedures of DNA extraction were prepared in a Class II biologic safety cabinet. DNA concentration was quantified using a NanoDrop 2000 spectrophotometer (Thermo Scientific, MA, USA). The integrity and size of DNA were assessed by 1% agarose gel electrophoresis.

The V3-V4 hypervariable region of the bacterial 16S rRNA gene was selected for polymerase chain reaction (PCR) amplification using universal primers (357F and 806R) linked with indices and sequencing adaptors. The primers also included the Illumina 5’ overhang adapter sequences for two-step amplicon library building. The detailed protocol of PCR reactions was described in Supplementary methods.

FASTQ sequences were uploaded to Basespace and reads were processed using BaseSpace Onsite 16 S Metagenomics App version 1.1.0 (Illumina). 16 S metagenomics data analysis used DNA from amplicon sequencing of prokaryotic 16 S small subunit rRNA genes with the high-performance version of Ribosomal Database Project Naïve Bayes algorithm [35]. The operational taxonomic unit (OTU) from the 16 S Metagenomics App was exported to a common-separated text file where the results were normalized by converting each OTU to a percentage of the reads from the sample. Any OTU with < 0.1% reads from a given sample were excluded. Resulting OTUs for each sample were utilized to construct relative concentrations of specific phylotypes. After assembling, full length sequences from paired ends were referenced against the Illumina curated version of Greengenes database (May 2013) at 97% identity level. Moreover, diversity indices were computed according to normalized amplicon sequence variant counts by a web-based platform (MicrobiomeAnalyst) [36]. As previously mentioned, microbial diversity was evaluated by alpha diversity (Shannon index, Simpson index, ACE, Chao 1) and beta diversity (Principle coordinates analysis, PCoA) in this study [37].

### DTI acquisition and analysis

MRI was acquired using a 3T GE Signa Excite scanner (GE Medical System, Milwaukee, WI, USA). DTI data were obtained using the following parameters: repetition time/echo time/flip angle = 9600 ms/62.7 ms/90°, a 192 × 192 mm^2^ field of view, b values of 1000 s/mm^2^ along 64 non-collinear gradient sensitizing directions, and with an isotropic voxel size of 2.2 mm. A null image with no diffusion weighted (b value = 0) was also acquired for DTI reconstruction. High resolution T1-weighted magnetization prepared rapid gradient echo imaging (MPRAGE) scans were obtained using the following parameters: repetition time/echo time/inversion time of 8600 ms/minimal/450 ms, a 256 × 256 mm^2^ field of view, and a 1-mm slice sagittal thickness with a resolution of 0.5 × 0.5 × 1 mm^3^ which cover the whole brain.

Data were preprocessed using a MATLAB toolbox called PANDA (Pipeline for Analyzing braiN Diffusion imAges), which was carried out through FMRIB Software Library (FSL 5.0.9, University of Oxford, UK) [38]. After correcting for the eddy current effect and motion artifacts by the FSL’s eddy tool, we used the FSL Brain Extraction Tool to remove non-brain regions from the corrected images [39]. Eventually, main diffusion metrics, including FA, MD, axial diffusivity, and RD, were calculated using FMRIB’s Diffusion Toolbox [40].

Subsequently, a combination of TBSS and atlas-based analysis was performed. PANDA first registered individual FA images of native space to the FMRIB58_FA template in the MNI space with spatial resolution of 1 × 1 × 1 mm^3^ and then applied the resultant warping transformations to write the images of other diffusion metrics. TBSS averaged all aMCI + subjects’ FA images and acquired a skeletonized image from this average FA. Then, this skeletonized image was projected to each subject’s standardized image. A FA threshold of 0.2 was used to exclude the voxels in gray matter and CSF during the TBSS processing procedure [20]. Finally, the regional average values of skeletonized images were obtained according to ICBM-DTI-81 WM labels atlas which divided the WM atlas into 50 brain regions. In this study, regions of interest were chosen based on previous model [16], including the fornix (column and body of fornix), bilateral cingulate gyrus (CG, cingulum), hippocampus (cingulum), and uncinate fasciculus.

### Statistical analyses

Results presented in our study were expressed as the numbers with percentages, means with standard deviation, as appropriate. Statistical analyses were performed by the IBM SPSS Statistics, version 25.0. Shapiro-Wilk test was used to examine the normal distribution of data, and we found that most variables were non-normal distributions. Demographic and clinical profiles of the HC and aMCI + groups were analyzed using Mann-Whitney U or chi-square test, when appropriate. Mann-Whitney U test was applied to compare alpha diversity among the different groups. Beta diversity was evaluated using PCoA and permutational multivariate analysis of variance (PERMANOVA) based on Bray-Curtis distance matrices. Differential abundance analysis was performed at each taxonomic level using Mann-Whitney test. The linear discriminant analysis (LDA) effect size (LEfSe) method was used to identify the taxa with statistical significance in the comparisons between aMCI + patients and HC, with an alpha value of 0.05 and LDA score of > 2 [41]. Relationship between dysbiotic microbiota, each NPI cluster, and limbic WM integrity in patients with aMCI + were explored using Spearman’s rank correlation, with Benjamini-Hochberg method for correcting multiple comparisons (false discovery rate was set at 0.05). To further examine whether the association of gut microbiota with NPI clusters was mediated by WM disruptions, a regression-based mediation analysis with the bootstrap method (5,000 bootstraps) was employed using Hayes’s PROCESS macro (SPSS model 4, version 4.1) [42]. In this model, only variables that exhibited a significant association with others were deemed the independent variable (gut microbiota), mediator (WM network), and outcome variable (NPI cluster). The mediation effect occurred when the direct effect of the independent variable on the dependent variable decreased with the inclusion of the mediator. A significant indirect effect was also obtained if the 95% confidence interval (CI) did not include zero. All paths were reported as unstandardized regression coefficients (β). Besides, our dataset had no missing data. The multicollinearity assumption was assessed using the variance inflation factor and tolerance values and the normal distribution assumption using residual normality, linearity, and homoscedasticity to satisfy assumptions of a regression-based analysis. The log transformation was applied to address skewed data. For all the statistical comparisons, p < 0.05 was considered as a significant level.

## Results

### Demographic and clinical characteristics

Table 1 shows the detailed demographic and clinical information of the HC and aMCI + groups. Both groups did not significantly differ in terms of age, gender distribution, years of education, body mass index, diabetes mellitus, constipation, or smoking status (p > 0.05). Most patients with aMCI + were treated with AD-related medicines, including donepezil and rivastigmine. Compared to HC, patients with aMCI + exhibited worse cognitive performance (p < 0.001). Patients with aMCI + had more depressive symptoms measured by Geriatric Depression Scale (p < 0.001), but they did not fulfill the diagnostic criteria for clinical depression.

**Table 1:** Demographics and neuropsychological assessments (mean ± SD)

	HC (n = 20)	aMCI + ^a^ (n = 20)	*P* value
**Demographic data**			
Gender (male)^b^	8 (40%)	12 (60%)	0.206
Age (years)	63.85 ± 7.74	67.05 ± 4.67	0.127
Education (years)	11.20 ± 3.04	10.80 ± 3.72	0.678
BMI	25.55 ± 3.41	23.73 ± 2.41	0.091
Diabetes mellitus^b^	6 (30%)	3 (15%)	0.256
Constipation^b^	1 (5%)	0 (0%)	0.311
Smoking^b^	1 (5%)	0 (0%)	0.311
*APOE* ε4^b^		6 (30%)	
Amyloid Centiloid score		35.20 ± 40.93	
Medication			
Donepezil^b^	0	12 (60%)	
Rivastigmine^b^	0	2 (10%)	
**Neuropsychological data**			
MMSE	29.40 ± 0.82	27.00 ± 1.81	< 0.001^*^
CASI	96.75 ± 2.66	88.37 ± 7.03	< 0.001^*^
CVVLT			
Total	29.65 ± 3.51	24.15 ± 4.91	< 0.001^*^
Delayed	8.80 ± 0.52	5.55 ± 2.67	< 0.001^*^
GDS	1.10 ± 3.08	2.85 ± 1.87	< 0.001^*^
NPI			
Total score		2.80 ± 2.65	
Behavioral composite score		0.20 ± 0.41	
Affective composite score		2.55 ± 2.68	
Psychotic composite score		0.05 ± 0.22	
DST			
Forward		7.70 ± 1.84	
Backward		4.50 ± 1.36	
VFT-animal		12.50 ± 4.40	
TMT-B (s)		71.85 ± 32.20	
FAQ		4.55 ± 5.07	
LQ		5.40 ± 0.99	

SD, standard deviation; HC, healthy control; aMCI+, amyloid-positive amnestic mild cognitive impairment; BMI, body mass index; *APOE* ε4, apolipoprotein ε4; MMSE, Mini-Mental State Examination; CASI, Cognitive Abilities Screening Instrument; CVVLT-total, Chinese Version Verbal Learning Test-total immediate recall; CVVLT-delayed, Chinese Version Verbal Learning Test-long delayed free recall (10 min); GDS, Geriatric Depression Scale; NPI, Neuropsychiatric Inventory; DST, Digit Span Test; VFT-animal, Verbal Fluency Test-animal; TMT-B, Trail Making Test-Part B; FAQ, Functional Activities Questionnaire; LQ, Lifestyle Questionnaire

^a^aMCI+ was defined by positive visual read out scores of 2 independent nuclear medicine physicians and not by the cutoff value of amyloid Centiloid scale

^b^Data are presented as n (%); ^*^p < 0.05

### Microbial diversity in aMCI + and HC

No statistical significance of alpha diversity was found between HC and aMCI + groups (Shannon index: U = 230.0, p = 0.429; Simpson index: U = 219.0, p = 0.620; ACE: U = 205.0, p = 0.904; Chao1: U = 204.0, p = 0.925) [Supplementary Fig. 1A]. The PCoA, based on the Bray-Curtis dissimilarity, also did not show significant differences between the two groups (F = 0.994, R^2^ = 0.025, p = 0.395) [Supplementary Fig. 1B].

### Disruption of gut microbiota in aMCI+

**Fig. 1:**
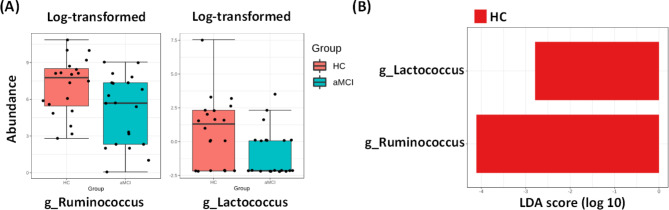
Differential abundance analysis of gut microbiota. (**A**) Mann-Whitney U test revealed significantly differential abundance of genera *Ruminococcus* and *Lactococcus* between aMCI + and HC groups. (**B**) LEfSe analysis showed significantly differential abundance of genera *Ruminococcus* and *Lactococcus* between aMCI+ (positive scores) and HC (negative scores) groups. Negative LDA scores indicated enriched taxa in the HC group (red). The LDA scores (log10) > 2 and *p* < 0.05 are listed. HC, healthy controls; aMCI+, amyloid-positive amnestic mild cognitive impairment; LEfSe, linear discriminant analysis (LDA) effect size

The significant alterations of gut microbiota between the HC and aMCI + groups were observed at the genus level [Fig. 1A]. Specifically, the relative abundance of genera *Ruminococcus* (p = 0.022) and *Lactococcus* (p = 0.031) was significantly lower in aMCI + group versus HC group. Given the above significant results, we further split the aMCI + group into High-aMCI + and Low-aMCI + groups according to the mean of amyloid burden. However, there were no significant differences in the relative abundance of genera *Ruminococcus* (p = 0.438) and *Lactococcus* (p = 0.485) between High-aMCI + and Low-aMCI + groups.

As shown in Fig. 1B, we also identified the distribution of certain microbiota at each taxonomic level between the two groups by LEfSe analysis. Compared to HC, patients with aMCI + exhibited significantly less abundance of genera *Ruminococcus* (LDA score = − 4.11, p = 0.021) and *Lactococcus* (LDA score = − 2.79, p = 0.030).

### Associations between gut microbiota, NPS, and limbic WM in patients with aMCI+

**Fig. 2:**
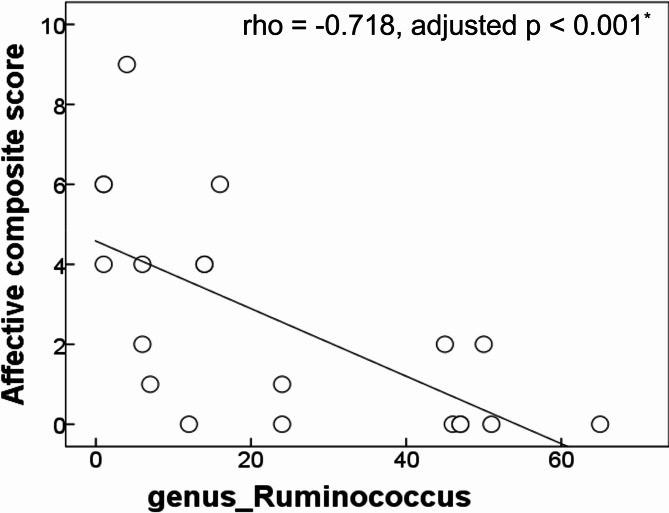
Significant association between the relative abundance of *Ruminococcus* and the affective composite score in patients with amyloid-positive amnestic mild cognitive impairment

Since significant between-group differences of relative abundance were detected in genera *Ruminococcus* and *Lactococcus*, we further investigated whether differentially abundant taxa were associated with the severity of NPS and limbic WM abnormalities in patients with aMCI+. Regarding the NPS, the relative abundance of genus *Ruminococcus* in aMCI + was negatively correlated with the NPI total score (rho = -0.667, p = 0.001). Based on this significant correlational result, we further divided the NPI into three clusters (i.e., psychotic composite score, behavioral composite score, and affective composite score). The relative abundance of genus *Ruminococcus* was negatively correlated with the affective composite score (rho = -0.718, adjusted p < 0.001) [Fig. 2]. However, there was no significant relationship between the relative abundance of genus *Lactococcus* and neuropsychiatric severity in patients with aMCI + after the corrections for multiple comparisons.

**Fig. 3:**
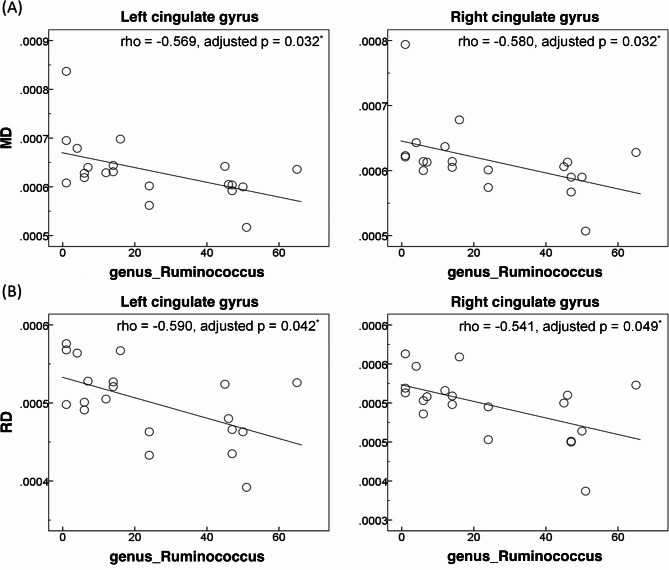
Significant associations between the relative abundance of *Ruminococcus* and white matter alterations (**A**, mean diffusivity [MD]; **B**, radial diffusivity [RD]) in the cingulate gyrus in patients with amyloid-positive amnestic mild cognitive impairment

In terms of the limbic WM integrity, the relative abundance of genus *Ruminococcus* in the aMCI + group was negatively correlated with MD value of the bilateral CG (left: rho = -0.569, adjusted p = 0.032; right: rho = -0.580, adjusted p = 0.032) and RD value of the bilateral CG (left: rho = -0.590, adjusted p = 0.042; right: rho = -0.541, adjusted p = 0.049) [Fig. 3A—B]. No other significant results were found after Benjamini-Hochberg corrections.

**Fig. 4:**
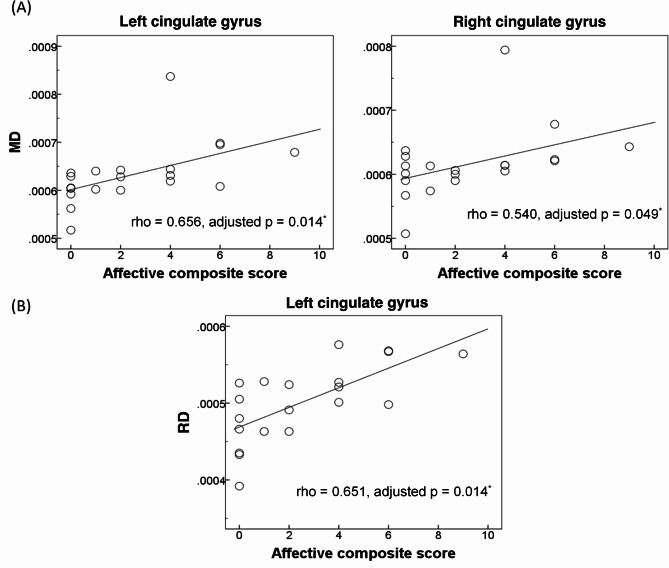
Significant associations between the affective composite score and white matter alterations (**A**, mean diffusivity [MD]; **B**, radial diffusivity [RD]) in the cingulate gyrus in patients with amyloid-positive amnestic mild cognitive impairment

The association of NPS with limbic WM aberration was further explored. The results showed that in the bilateral CG, the affective composite score was positively correlated with MD value (left: rho = 0.656, adjusted p = 0.014; right: rho = 0.540, adjusted p = 0.049) and RD value (left: rho = 0.651, adjusted p = 0.014) [Fig. 4A—B]. No other significant results were obtained after Benjamini-Hochberg corrections.

### The mediating role of the left cingulate gyrus in the relationship between Ruminococcus and the severity of affective impairment

**Fig. 5:**
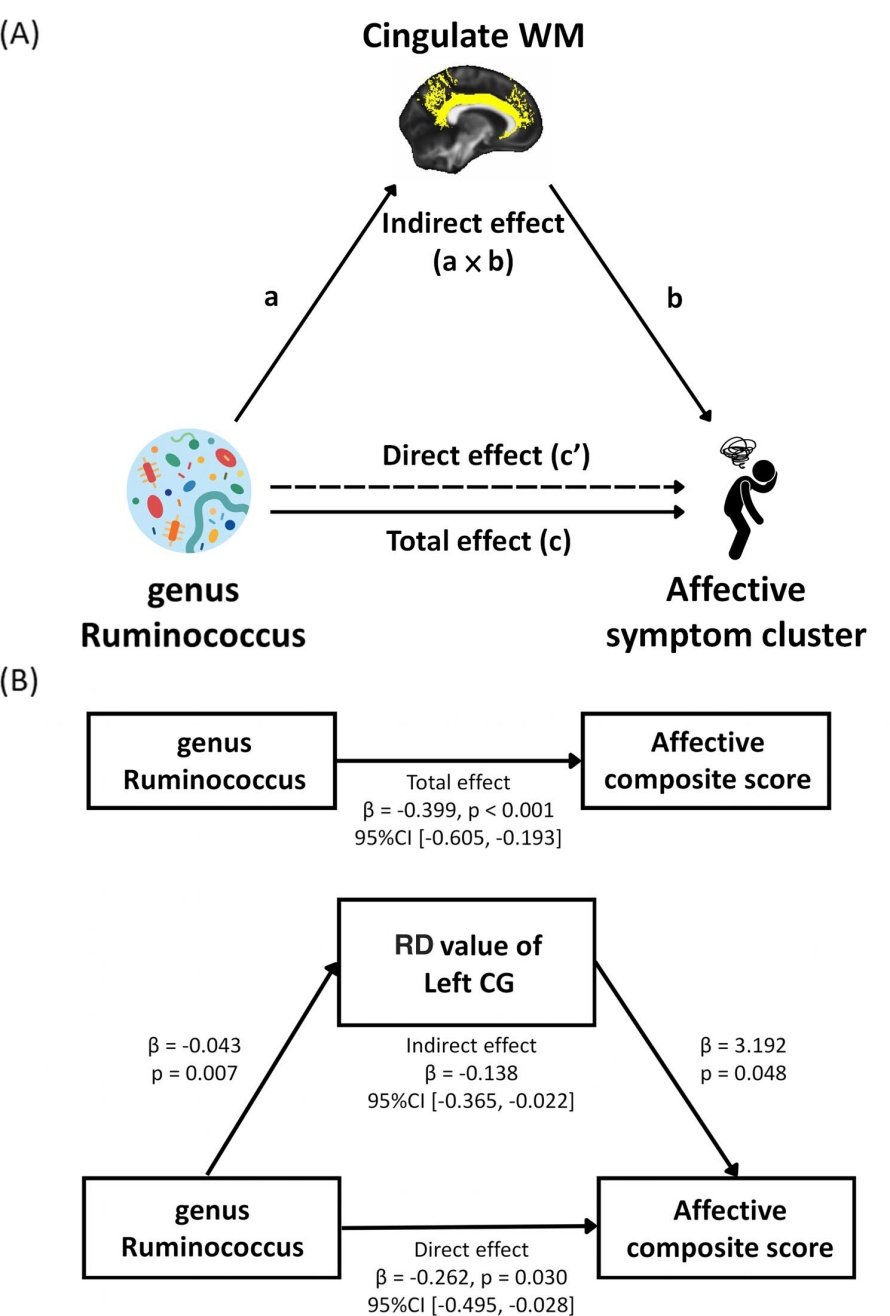
(**A**) Mediation model in patients with aMCI+. (**B**) Mediation analysis between the relative abundance of *Ruminococcus* and the affective composite score, with RD value of the left CG as the mediator aMCI+, amyloid-positive amnestic mild cognitive impairment; WM, white matter; RD, radial diffusivity; CG, cingulate gyrus; CI, confidence interval

Mediation analysis revealed that the total effect of genus *Ruminococcus* on the severity of affective symptom cluster was significant in patients with aMCI+ (β = -0.399, p < 0.001). When WM connectivity of the left CG (RD value) was introduced to the model as a mediator, the direct effect remained significant with a decreased magnitude (β = -0.262, p = 0.030), suggesting the impact of genus *Ruminococcus* on the affective composite score was a partial mediation effect. The indirect effect of WM connectivity in the left CG (RD value) was also significant (β = -0.138, 95% CI [− 0.365, − 0.022]) [Fig. 5]. Additionally, after including the mediator of WM integrity in the bilateral CG (MD value) to the model, we did not observe the mediation effect in these two models (indirect effect = left: β = -0.084, 95% CI [− 0.303, 0.027]; right: β = -0.044, 95% CI [− 0.196, 0.072]) (Supplementary Fig. 2).

## Discussion

To the best of our knowledge, this is the first study to comprehensively explore the impact of gut dysbiosis on NPS and limbic WM connectivity in patients with aMCI+. The main findings of this study were three-fold. Firstly, despite no significant shifts in microbial diversity between the HC and aMCI + groups, the relative abundance of genera *Ruminococcus* and *Lactococcus* was markedly decreased in patients with aMCI + versus HC. Secondly, reduced abundance of *Ruminococcus* was associated with higher severity of the affective symptom cluster and more disrupted WM integrity in the bilateral CG among patients with aMCI+. Finally, the left CG was a significant mediator of the relationship between *Ruminococcus* and mood disturbance.

The genus *Ruminococcus*, belonging to phylum *Firmicutes*, was significantly reduced in patients with aMCI + as compared to HCs. This strain is one of gram-positive bacteria and the producer of propionate, serving a connection with glucose homeostasis and inhibition of cholesterol synthesis [43]. It has been shown that this dysbiotic microbiota would impair the permeability of gut and blood-brain barrier, which promotes the dissemination of intestinal amyloid into the systemic circulation and then deposits in the brain [44]. Besides, several lines of evidence have confirmed that the depleted *Ruminococcus* is observed in the MCI or dementia stage of AD [15, 44–46], which supports our current findings. A recent study further showed that the level of *Ruminococcus* was elevated in the mice with ochratoxin A-induced cecal injury after probiotic treatment [47]. Therefore, this genus of bacteria is a beneficial strain for humans.

Decreased abundance of genus *Lactococcus* was also observed in patients with prodromal AD. This genus from phylum *Firmicutes* has been shown to participate in the production of L-lactic acid from glucose fermentation and the activation of mucosal function and systematic immunity [48]. Prior evidence has revealed that lactic acid prevents neurons from mitochondrial damage caused by the amyloid deposition in AD [49]. Notably, this dysbiotic microbiota may lead to impairments in brain functions, such as decrease in synaptic remodeling, axonal excitability, and neural oxidative metabolism [50]. Furthermore, *Lactococcus lactis* (genus *Lactococcus*) has been found to be implicated in the biosynthesis of nicotinamide mononucleotide, which is relevant to anti-aging [51]. Intriguingly, previous studies have discovered that patients with AD manifest lower abundance of *Lactococcus* than HC [52, 53], aligned with our results found in patients with aMCI+. Hence, this microbial strain may have an advantageous effect on host physiology.

This study showed that WM integrity in the left CG played a partial mediating role in the relationship between the relative abundance of *Ruminococcus* and the severity of affective symptom cluster in patients with aMCI+. It has been proposed that the effects of the gut microbiota on affective impairments was modulated by microbe-derived neurotransmitters and metabolites [54]. Especially, 5-hydroxytryptamine (5-HT, serotonin) plays a key role in emotion regulation. Once 5-HT is released by enterochromaffin cells, it can cross the intestinal epithelium and the blood–brain barrier via the circulatory system to reach the brain [55]. Furthermore, *Ruminococcus* has been reported to participate in tryptophan metabolism, which is associated with the biosynthesis of tryptamine and serotonin [56]. More specifically, tryptamine produced by specific bacteria (e.g., *Ruminococcus*) is a neuromodulator between the excitatory and inhibitory activity of serotonin; tryptophan is the precursor of serotonin, which is widely discussed for its entangled relationship with mood disorders. Previous studies have shown that reduced circulating concentrations of tryptophan would functionally affect emotions using acute tryptophan depletion protocol [57, 58]. Similarly, it has been found that the relative abundance of *Ruminococcus* is positively correlated with the levels of serotonin [59], supporting a link between the abundance of *Ruminococcus* and the severity of affective symptoms in our patients. Data from animal models further demonstrated that dietary interventions alleviated depressive symptoms, as shown by the enriched abundance of *Ruminococcus* and improvement in depression-induced tryptophan reduction [60]. Therefore, targeting the gut microbial tryptophan metabolism by regulating the endogenous gut microbiota or changing the dietary pattern may indicate alternative strategies to ameliorate mood disorder.

On the neurochemical level, emotional distress in patients with AD is relevant to serotonergic denervation in various brain regions, including the limbic area [61–63]. Of note, the CG has consistently been demonstrated a reduction of serotonin transporters or receptors in patients with MCI, which is closely tied to emotion and other cognitive functions. Neuroimaging research showed that aMCI patients with higher levels of apathy exhibited increased MD values in the cingulate region [64]. Likewise, it was reported that the CG lesions along with sleep disorders and apathetic symptoms were observed in those with aMCI or dementia [65, 66]. Moreover, previous literature has indicated that the CG mediates the function of the HPA axis through its projections to the hypothalamus [67]. It should be noted that cingulate dysfunction is associated with the dysregulation of HPA axis. The overactivation of HPA axis would lead to decreased 5-HT receptors binding and increased concentrations of corticosterone [68]. More importantly, the altered gut microbiota may affect the serotonergic system. Evidence from clinical trials has revealed that lower abundance of *Ruminococcus* in depressive individuals is concomitant with reduced levels of blood tryptophan and serotonin in the CG [67, 69]. Taken together, our findings suggest that the gut microbiota may be a promising target for mitigating the affective symptoms in patients with aMCI+.

The present study has several limitations. First, we cannot fully disentangle physiological interaction between the dysbiotic microbiota (i.e., genera *Ruminococcus* and *Lactococcus*) and disease pathologies due to the observational design of the present study. Consequently, these altered strains merit future studies with a longitudinal design to have an in-depth exploration. Second, we could not confirm that HC were amyloid-negative because this group did not have amyloid imaging. Finally, despite the lack of measuring WM integrity in HC, we still reasoned that the disrupted patterns of limbic bundles in aMCI + resulted from AD pathogenesis, as evident by the strict diagnostic criteria of the disease. In the future, we hope to administer the HC group with DTI scans, which may provide more accurate proof of the relationship between the dysbiotic taxa and WM lesions in patients with aMCI+.

## Conclusions

This study provides evidence that the gut dysbiosis can influence limbic microstructure in patients with aMCI+. Besides, WM integrity in the left CG may be a partial mediator of the association between genus *Ruminococcus* and affective symptoms, supporting that the MGB axis can conjointly affect emotional behaviors.

### Electronic supplementary material

Below is the link to the electronic supplementary material.

Supplementary Material 1

## Data Availability

Raw FASTQ sequences generated from the 16S amplicon sequencing of fecal DNA are available in the NCBI Sequence Read Archive under Accession Number PRJNA988280 (http://www.ncbi.nlm.nih.gov/bioproject/988280).
