# Supplementary material for: Cingulate white matter mediates the effects of fecal Ruminococcus on neuropsychiatric symptoms in patients with amyloid-positive amnestic mild cognitive impairment
Source: BMC Geriatr. 2023 Nov 7;23:720. doi: 10.1186/s12877-023-04417-9 (PMC10631051; doi:10.1186/s12877-023-04417-9)
Supplement: Supplementary file 1 — Supplementary Material 1 [file 12877_2023_4417_MOESM1_ESM.docx]

**Supplementary methods**

**Amyloid PET**

The amyloid tracer ^18^F-florbetapir was synthesized at the cyclotron facility of Chang Gung Memorial hospital. Brain positron-emission tomography (PET) scans were obtained after the injection of 296 ± 74 MBq by using a GE discovery MI PET/ computed tomography scanner. The acquisition protocol, optimal scanning time and image reconstruction followed a previous study (Chang et al., 2016). Amyloid positivity was first confirmed by positive read outs of two nuclear medicine physicians that were blinded to the clinical status.

**Amyloid Centiloid calculations**

For quantification, we calculated the amyloid Centiloid scale followed that described in the Centiloid project (https://www.gaain.org/), the equations by Klunk et al. (2015), SPM12 (https://www.fil.ion.ucl.ac.uk/spm/software/spm12/) unified segmentation method on MATLAB2019b (MathWorks, Natick, MA, USA) and Mac OS workstation with Catalina Version 10.15.6.

In brief, the 10-minute 5 mm filtered PET frames were registered to corresponding 3D MPRAGE scans and transformed to the Montreal Neurological Institute (MNI) 152 space. We calculated the standardized uptake value ratios (SUVr) in MNI space using the target region provided in the GAAIN website (https://www.gaain.org/). Amyloid SUVr were calculated by dividing the voxel number weighted average of mean uptake of the template mask (prefrontal, orbitofrontal, parietal, temporal, anterior and posterior cingulate and the precuneus) by the uptake of the whole cerebellum. Mean SUVr values were calculated for all cases.

The transformation of our tracers (^18^F-florbetapir ^FBP^SUVr) to the reference (^11^C-PiB ^PiB^SUVr) was performed using linear regressions and the resulting conversion from ^18^F-florbetapir to ^11^C-PiB: ^PiB^SUVr = 1.6911 ^FBP^SUVr - 0.7234 (R^2^ = 0.889). In our case, the final equation resulted as: CL = 100(^PiB^SUVr - 1.011) / (2.056 - 1.011). When we compared the resultant Centiloid values against the ones published, we found a high correlation (R^2^ = 0.993) with all the criteria (Slope = 0.998, Intercept = 0.1255 CL) falling within the recommended range (R^2^ > 0.98, Slope = 0.98 to 1.02, Intercept = -2 to 2 CL). The results were within the 2% range of the original methods (1.01 in the controls and 2.076 in the patients with Alzheimer's disease).

Chang, Y. T., Huang, C. W., Chen, N. C., Lin, K. J., Huang, S. H., Chang, W. N., Hsu, S. W., Hsu, C. W., Chen, H. H., & Chang, C. C. (2016). Hippocampal Amyloid Burden with Downstream Fusiform Gyrus Atrophy Correlate with Face Matching Task Scores in Early Stage Alzheimer's Disease. *Front Aging Neurosci*, *8*, 145. https://doi.org/10.3389/fnagi.2016.00145

Klunk, W. E., Koeppe, R. A., Price, J. C., Benzinger, T. L., Devous, M. D., Jagust, W. J., Johnson, K. A., Mathis, C. A., Minhas, D., Pontecorvo, M. J., Rowe, C. C., Skovronsky, D. M., & Mintun, M. A. (2015). The Centiloid Project: Standardizing quantitative amyloid plaque estimation by PET. *Alzheimers & Dementia*, *11*(1), 1-15. https://doi.org/10.1016/j.jalz.2014.07.003


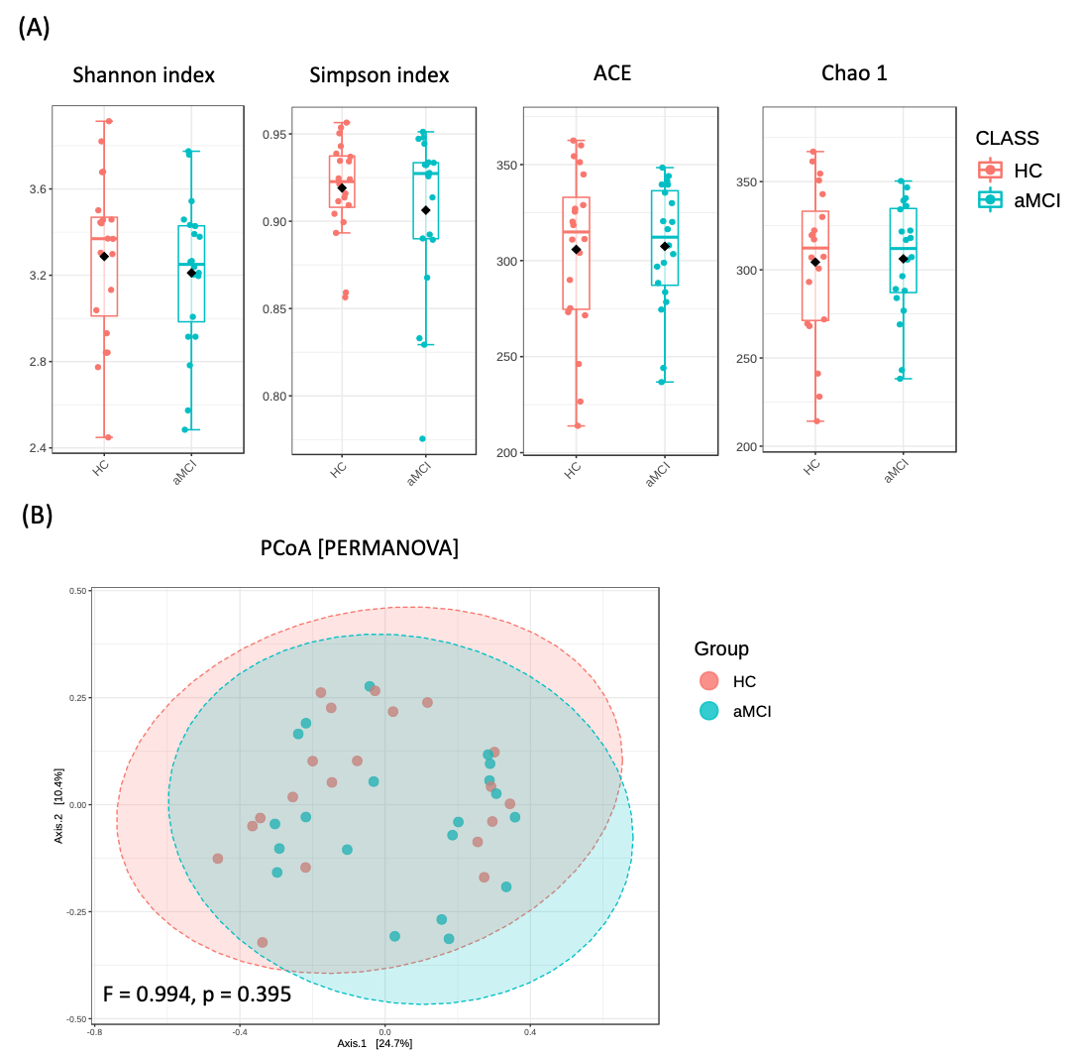


**Supplementary Figure 1**. Microbial diversity. (A) The alpha diversity between aMCI+ and HC (Shannon index, Simpson index, ACE, and Chao1). Each bar graph represents the median, interquartile range, minimum, and maximum values. (B) The beta diversity between aMCI+ and HC (PCoA based on the Bray-Curtis metric). HC, healthy controls; aMCI+, amyloid-positive amnestic mild cognitive impairment; PCoA, principal coordinate analysis; PERMANOVA, permutational multivariate analysis of variance.


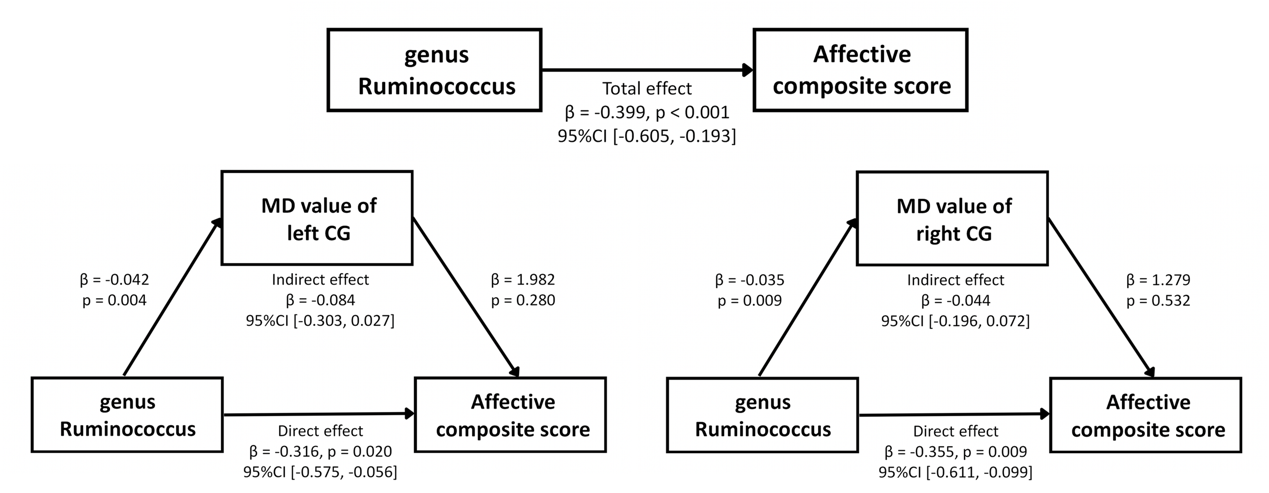


**Supplementary Figure 2.** Mediation analyses between the relative abundance of Ruminococcus and the affective composite score, with MD value of the bilateral CG as the mediators in individuals with aMCI+. aMCI+, amyloid-positive amnestic mild cognitive impairment; MD, mean diffusivity; CG, cingulate gyrus; CI, confidence interval.
